# Book Reviews

**Published:** 1888-12

**Authors:** 


### Book Reviews.

**Hand-Book of Historical and Geographical Phthisiology**; with special reference to the distribution of consumption in the United States. Compiled and arranged by George A. Evans, M. D., Member of the Medical Society of the County of Kings, New York; Member of the American Medical Association, etc. 12mo, pp. 295. New York: D. Appleton & Co. 1888.

In compiling the above work, Dr. Evans has rendered a most useful service to the profession. Heretofore, it has been necescary to glean from many sources the knowledge here contained in one small volume. Any one who has had occasion to perform this arduous work will appreciate at once the labors of the author. The task he had before him is well stated in the preface: "In the following volume I have attempted to present a sketch of the development of our knowledge of pulmonary consumption from the time of Hippocrates up to the present day, together with the ascertained facts regarding the geographical distribution of that affection. It has also been my effort so to arrange the statistics in regard to the geographical distribution of consumption in the United States as to make them available for convenient reference in selecting localities of resort, or residence for invalids, and also for those who are in health." This task has been performed thoroughly and well. The divisions of the book are as follows: I. Historical Sketch. II. Geographical Distribution of Consumption in Countries other than the United States. III. Geographical Distribution of Consumption in the United States. IV. Topography and Climate of States and Territories, and Summary for States, Groups, Cities, and for Counties of 10,000 Population and upward, showing the Number of Deaths from Consumption per 1,000 Inhabitants. V. Meteorology. VI. Etiology. VII. Conclusions.

It will be seen at once that the author handles the subject in a comprehensive way. We feel sure the book will be found upon the table of every physician interested in this widespread and fatal disease. We notice the author says, and justly, that New York State is noted for the beauty of its lakes, and he then proceeds to name the principal ones. "In the west are Chautauqua and Cattaraugus." We have never heard of the latter, nor can we find it upon any map. Whatever Cattaraugus county is famous for—and we feel sure it is famous for something—it cannot lay claim to any body of water large enough to be dignified with tbe name of a lake. The author has been so careful in his statements, that even a slight inaccuracy like this should be pointed out, so that in the future it may be corrected.

---

**The Ear and Its Diseases:** Being Practical Contributions to the Study of Otology. By SAMUEL SEXTON, M. D., Aural Surgeon to the New York Eye and Ear Infirmary; Fellow of the American Otological Society; Fellow of the New York Academy of Medicine; Member of the Medical Society of the County of New York, and of the Practitioners' Society of New York. Edited by CHRISTOPHER J. COLLES, M. D. Octavo, 473 pages. Numerous illustrations. Extra muslin, $4.00. New York: William Wood & Co.

It is the pleasant part of the reviewer's task to praise work well done. This cheerful duty is ours in calling attention to the work noticed above. Dr. Sexton has written a book which merits the description of its title page. It *is* a book of "Practical Contributions to the Study of Otology." The author is a teacher of long experience, and he has had large opportunities to make and correct observations upon the diseases of the ear. These observations and their lessons, or, at least, a good part of them, he presents to us in this book. It is not a text-book for students, though they can use it to advantage; it is rather a work for the physician who has already had experience in treating the diseases of the ear, and who desires to add to his knowledge by familiarity with the experience of others. The author tells us in the preface that many subjects are considered here that are not usually prominent in works upon the ear, and this statement is borne out by the study of its pages. Among these are the following: Catarrh of the Upper Air Tract; Oral Irritation, especially Dentition and Deceased Teeth; Sea Bathing; Wounds and Injuries of the Ear, occurring in warfare and civil life; Rupture of the Drum-head from Boxing the Ears; Concussion from the Blast of Great Guns, Explosives, etc.; Anomalies of Audition; The Effects of False Hearing on Singers, Lecturers, Actors, and Musicians; Othematoma occurring in Lunatics, Pugilists, and others.

There is a chapter devoted to "The Operation of Excision of the Drum-head and Ossicles for Otorrhea and Deafness due to Catarrh of the Middle Ear." It is stated that the results of this operation have been satisfactory, and that it is hoped that its usefulness will be confirmed by experience.

Another chapter considers the subject of "Defective Hearing among School Children and Teachers." This is a very important subject, and this chapter merits careful study by every physician.

The author's observations on "The Effects of High Atmospheric Pressure on the Ear, in Tunnels, Caissons," etc., already familiar to the profession, has been incorporated and forms a chapter. Finally, we have here considered the claims of soldiers, sailors, and marines, for pensions on account of disability from deafness. Altogether this work shows the great advance that has been made in otology during the past few years, and it also shows that the author has contributed his share to this advance.

A word must be said about the work of the publishers. The book is well printed, the type clear, and the illustrations, many of which are original, are very fine. This, of course, adds to the attractiveness of the work and to the pleasure of those who will follow the author through its instructive pages.

---

**The Physician's Leisure Library.** Clinical Lectures on Certain Diseases of the Nervous System. By Professor J. M. CHARCOT, Professor to the Faculty of Medicine, Paris, France; Physician to the Salpetriere; Member of the Institute and Academy of Medicine; Honorary President of the Anatomical Society, etc. Translated by E. P. HURD, M. D. Member of the Massachusetts Medical Society and of the Climatological Society, Newburyport, Mass. 1888: George S. Davis, Detroit, Mich.

The writings of Charcot, and especially these lectures, have been fully appreciated by the general practitioner, as well as the specialist, who reads French. The opportunity for a greater number to profit by them is now given through the efforts of Dr. Hurd, who has given us a good translation of eight lectures delivered by the author. The publisher has done his part, and the work is presented in a neat form. The chapter on Spiritism and Hysteria is very interesting. It recites of The Influence of Intellectual Excitations on the Development of Hysteria; Belief in the Supernatural, of the Marvelous; Practice of Spiritism; Account of an Epidemic of Hysteria in a family of three childdren inhabiting a Military Penitentiary and Addicted to Spiritism; Concerning antecedent Nervous and Rheumatic attacks; Permanent and variable Stigmata. Under the head of Treatment, he points out the necessity of isolation, suppression of all visits on the part of parents and friends; of modifying the diathesis, if any exist (rheumatic, for instance); of static electrization, methodical hydrotherapy, etc. The other lectures are on Choreiform Movements and Tremblings, Rythmical Chorea. He treats of the Trembling of Multilocular Sclerosis, of Paralysis Agitans, Senile Tremor, Vibratory Tremblings, Tremor of General Paralysis, of Basedow's Disease, Chorea, Characters of the Involuntary Movements of Sydenham's Chorea, Chorea and Hemi-Chorea, Pre- and Post-Hemiplegic, Athetosis and Hemi-Athetosis, Rythmical Chorea. The third chapter is on Muscular Atrophy consequent on certain Articular Lesions, calling attention to the fact that the intensity of the joint lesion is not in proportion to the paralytic and atrophic phenomena. So on throughout this little work there is much information in a very condensed form.

---

**Annual Reports of State Boards of Health.** Pennsylvania, Second; 1886. Michigan, Fifteenth; 1887. New York, Eighth; 1888.

The Second Annual Report of the State Board of Health and Vital Statistics of the Commonwealth of Pennsylvania is a volume of pp. 1056, handsomely bound in tan muslin, with red edges. It embraces, besides the report of the Secretary and the minutes of the proceedings of the Board at its several sessions during the year, voluminous appendices of reports by standing committees, sanitary inspectors, proceedings of sanitary conventions, scientific papers on public health problems, and correspondence with other boards, officers, sanitarians, etc., etc.

Owing to failure on the part of the Legislature to grant permission for the printing of the report, it has been delayed beyond the usual time for its appearance, a fault which the Board hopes to correct in future. The report reflects great credit upon Dr. Benjamin Lee, the efficient secretary of the Board, who has edited the volume with great care. The report is accompanied by a compendium of the Laws Relating to Public Health and Safety of the State of Pennsylvania, in pamphlet form.

The Fifteenth Annual Report of the Secretary of the State Board of Health of Michigan is a less pretentious volume than that of Pennsylvania, but contains much that is valuable to the sanitarian, public health officer, and physician.

The two papers relating to Tyrotoxicon, the recently discovered milk-poison, by Prof. Victor C. Vaughn, M. D., are well worth careful examination. The Secretary, Dr. Henry B. Baker, of Lansing, who has become recognized authority on public health and sanitary questions, always presents a useful and clear report, embracing not only matters pertaining to his own State, but also much of general interest to sanitarians in other regions of the country.

The Eighth Annual Report of the State Board of Health of New York, though containing a few valuable sanitary papers, is not such a volume as the Empire State should put forth as its annual contribution to public health science. We impute no dereliction of duty to the members of the Board, nor lack of skill in sanitary science, but consider it rather the fault of the legislative authorities, who make such meager provision for scientific work. Even the young State of Nebraska, with its well-equipped Patho-Biological Laboratory, is doing more to foster scientific research than the great State of New York, with its six millions of people, possessed of untold wealth and the highest cultivation. Let us hope that our legislators will spare a little of the time that is annually given to "practical politics," and devote it to the consideration of matters concerning public health, supporting considerate action with liberal appropriations, to the end that our State may assume its proper place in the front rank of progress, and in all that concerns the welfare of its citizens.

---

**Second Series (Complete in twelve parts)—Photographic Illustrations of Skin Diseases, an Atlas and Text-Book combined.** Parts VII. and VIII. By George Henry Fox, A. M., M. D., Clinical Professor of Diseases of Skin, College of Physicians and Surgeons, New York. Hand college plates—nearly one hundred cases from life. New York: E. B. Treat, No. 771 Broadway.

The first series of this valuable work received so favorable a reception from the profession that the publisher, with commendable enterprise, ventures the issue of the present series. We have a full appreciation of the value of the work, having it in our library, and resorting to it as an aid in diagnosis and treatment.

Part VII. illustrates in its plates Pemphigus, Acne Vulgaris, Porrigo (impetigo contagiosa), Purpura.

Part VIII. furnishes plates illustrating Cornea Cutanea, Keratosis, Fothcularis, Ichthyosis, Elephantiasis and Rosacia.

We commend the work to the profession for its accuracy of illustration and for the practical treatment the text furnishes.

---

**Atlas of Venereal and Skin Diseases.** Comprising original illustrations and selections from the plates of Prof. M. Kaposi, of Vienna, and others. With original text by Prince A. Morrow, A. M., M. D., Clinical Professor of Venereal Diseases in the University of the City of New York. Fasciculi VII. and IX. New York: William Wood & Company. 1888.

In the brief space allotted, it will be impossible to give a suitable review of these admirable plates and the text accompanying them. We are content to give the list of illustrations in each part, and to say that in accuracy of delineation, and in fullness of description, this work surpasses any kindred one we have had presented to us for examination. The seventh fasciculus furnishes five plates, ranging from XXXI. to XXXV. ¡nclusive.

Plate XXXI.—Figure I. Ulcerative gumnata. Figs. II. and III. Vegetating syphilides of the face and soft palate.

Plate XXXII.—Figs. I. to XV. Syphilis of the mucuous membrane of the lips, the tongue, the hard and soft palate, the pharynx.

Plate XXXIII.—Figs. I. and IV. Paronychia and onychia syphilitica. Figs. II. and III. Ulcerative syphilides of the nose. Figs. V. and VI. Ulcerating Syphilides of Commissure of Ibes.

Plate XXXIV.—Fig. I. Syphilitic Pemphigus. Fig. II. Polymorphous Syphilide (inherited).

Plate XXXV.—Figs. I., II., III. Maculo-Papulæ, and other forms of inherited syphilis. Figs. IV., V., VI., VII. Syphilitic Pemphigus of the palms and soles.

Fasciculus IX. contains five plates as follows:

Plate XLI.—Erythema Marginatum, Erythema Papulatum, Erythema Tris et Circinatum.

Plate XLII.—Herpes Iris. Erythema Nodosum.

Plate XLIII.—Urticaria, Urticaria Pigmentosa.

Plate XLIV.—Eczema Capitis, Eczema Faciei.

Plate XLV.—Eczema Papulosum, Vesiculosum, et Impetigenosum. Eczema Squamosum.

The plates plainly illustrate this obscure class of diseases, and are a valuable aid to the practtioner in their diagnosis and treatment.

---

**The Physician's Pocket Day-Book.** Designed by C. HENRI LEONARD, M.A., M. D. Size, 7½ inches long, 3½ inches wide, and ⅜ of an inch thick. Bound in red morocco, for the pocket; pencil loop and flap, red edges. Price, $1.00 postpaid. The Illustrated Medical Journal Co., publishers, Detroit. 1888.

This is the tenth year of issue of this exceedingly popular day-book, which contains several new features. Besides accommodating daily charges for thirteen months for fifty families, and the other usual memorandum pages, it has a very complete list of doses of old and new drugs; poisons and their antidotes, tried tests for urinary deposits, chemical and microscopical; obstetric calendar; disinfectants for the sick room and vaults; tables of weights and measures; table of eruptive fevers, and drops in a drachm of fluid medicines. Dr. Leonard has certainly devised a very useful record, that can be used either as an account-book or a visiting list, and can be opened at any date.

---

**Quiz Compends.** No. 8. A Compend of the Diseases of the Eye, including Refractions and Surgical Operations. By L. WEBSTER FOX, M. D., Opthalmic Surgeon to the Germantown Hospital, etc., etc., and GEORGE M. GOULD, M. D. Second edition, revised and enlarged, with seventy-one illustrations. Philadelphia: P. Blakiston, Son & Co., No. 1012 Walnut street. 1888.

These quiz compends are issued to supply the medical student with the salient points in the diagnosis and treatment of ocular disorders, and to furnish the general practitioner with a few outlines of the science of which they treat. They really endeavor to compass a résumé of the subject within certain limits, and are a valuable aid to the student who strives to "coach" for his examinations. This compend is the most valuable of its kind we have received, and fully fulfills the objects its author has in view in its preparation.

**The Physician's Visiting List (Lindsay & Blakiston's) for 1889.** Thirty-eighth year of its publication. Philadelphia: P. Blakiston, Son & Co.

This standard visiting list is one of the best published, and always an acceptable one to the busy physician. For a statement of its special features we refer the reader to our advertising columns.

